# Insomnia Symptoms and Self-Regulated Eating Behavior in Hispanic Emerging Adults: An Exploration of Acculturative Stress

**DOI:** 10.3390/brainsci15080780

**Published:** 2025-07-22

**Authors:** Ainsley A. Miller, Pablo Soto, Mayra S. Ramos, Spencer A. Nielson, Natalie Dautovich, Rosalie Corona, Joseph M. Dzierzewski

**Affiliations:** 1 Department of Psychology, Virginia Commonwealth University, Richmond, VA 23284, USA; milleraa4@vcu.edu (A.A.M.); sotop2@vcu.edu (P.S.); ramosms@vcu.edu (M.S.R.); nielsons@vcu.edu (S.A.N.); ndautovich@vcu.edu (N.D.); racorona@vcu.edu (R.C.); 2 National Sleep Foundation, 2001 Massachusetts Ave NW, Washington, DC 20036, USA

**Keywords:** sleep, eating, insomnia symptoms, acculturative stress, emerging adults

## Abstract

**Background/Objectives**: Research regarding the relationship between insomnia symptoms and self-regulated eating behaviors in Hispanic populations is limited, particularly that pertaining to emerging adulthood (18–25 years old) and the potential role of cultural factors. The present study explored the association between insomnia symptoms and self-regulated eating behaviors in a Hispanic emerging adult sample, as well as the role of acculturative stress. **Methods**: Participants who identified as Hispanic between the ages of 18–25 years old and were English-speaking completed an online survey assessing insomnia symptoms, self-regulated eating behavior, and acculturative stress. **Results**: In a multiple regression analysis, insomnia symptoms emerged as a significant predictor of self-regulated eating behavior such that Hispanic individuals with increased insomnia symptoms were more likely to report low self-regulated eating behavior. Furthermore, it was found that acculturative stress partially mediated the association between insomnia symptoms and self-regulated eating behavior. **Conclusions**: Insomnia symptoms are a risk for problematic eating behaviors in Hispanic emerging adults. Identifying other early risk factors, including culturally unique risks like acculturative stress, may be important for the development of tailored early intervention efforts to reduce problematic eating patterns.

## 1. Introduction

Emerging adulthood (i.e., 18–25 years of age) is marked by significant physiological, psychological, and life changes that impact health behaviors, such as increased risk-taking behaviors, changes in metabolism and physical activity, and increased mental health concerns [1,2,3,4]. All of these changes are known to be associated with sleep [5,6]. Insomnia symptoms, in particular, can impact aspects of health including eating behavior and mental health [7,8,9]. Yet relatively few studies have examined the association between insomnia symptoms and self-regulated eating behaviors, especially in historically underserved populations like Hispanic emerging adults. This is unfortunate given the fact that Hispanic individuals face elevated risks for conditions such as obesity and diabetes, which are linked to eating behaviors, and represent one of the fastest growing demographic groups in the US population.

Increased social independence and risk-taking behavior in emerging adulthood has been shown to impact eating regulation, dietary quality, and compulsive and emotional eating [10,11]. Self-regulated eating is defined as the ability to consciously manage and control food intake, and represents an important step between eating intention and eating behaviors [12]. Emerging adulthood also presents many challenges to sleep, including high rates of insomnia symptoms and insufficient sleep duration [6]. Interestingly, research supports a bidirectional relationship between sleep and eating behaviors [7,13,14,15]. For example, people with short sleep are more likely to have lower quality nutrient intake, nocturnal eating, and emotional and uncontrolled eating [7,14,15,16,17]. Similarly, people with high fat diets are more likely to experience non-restorative sleep [7]. Relatively little is known about the important connection between insomnia symptoms and self-regulated eating behaviors in Hispanic individuals.

Both sleep and eating behaviors may differ among racial ethnic groups. Hispanic individuals are more likely than non-Hispanic Whites to report short sleep, disrupted sleep, and decreased sleep quality [18,19,20]. With regard to eating behavior, among Hispanic populations problematic eating often includes binge eating, meal skipping, emotional and uncontrolled eating, and diuretic use [16,21,22,23], and Hispanic individuals appear less likely to seek treatment for their problematic eating [24]. Insomnia symptoms in Hispanic emerging adults are related to changes to BMI and poorer perceived physical health [25]. Disrupted eating behaviors are predicted by insomnia symptoms in Hispanic adults [17]. Whether culture-specific factors may influence the association between insomnia symptoms and self-regulated eating in Hispanic individuals is unknown.

One particularly salient cultural factor that may affect both sleep and eating behaviors among Hispanic emerging adults is acculturative stress. Acculturative stress is defined as the reaction to the pressure of acclimating to a dominant culture, as well as the stress related to the reconciliation with both ethnic and mainstream culture [26,27]. Though limited research exists to date, some studies within Hispanic samples point to the meaningful role of acculturative stress for both sleep and eating behaviors, including a negative correlation between acculturative stress and sleep duration [28] and acculturative stress and emotional eating, eating pathology, and BMI [29,30].

The purpose of the present study was to examine the associations among insomnia symptoms, acculturative stress, and self-regulated eating behaviors in a Hispanic sample of emerging adults. Specifically, we aimed to replicate and extend previous work [16], which identified an association between poor sleep and eating behaviors in a Hispanic adult sample, to a sample of Hispanic emerging adults. Further, we aimed to determine if acculturative stress mediates the association between insomnia symptoms and self-regulated eating behaviors in Hispanic emerging adults. We hypothesize that increased insomnia symptoms would be associated with lower self-regulated-eating behavior in Hispanic emerging adults and that acculturative stress would partially mediate this relationship.

## 2. Methods

### 2.1. Procedures

Individuals were recruited to participate in the Sleep in Emerging Adulthood and Stress Response to Acculturation (SIESTA) study, which focused on sleep in Hispanic emerging adults. Data collection occurred between October 2020 and February 2022. Recruitment largely took place at a large public university in the southeastern United States and through social media. All potential participants were sent a weblink to complete study paperwork. Individuals interested in participating in the study were screened for inclusion and exclusion criteria, which included being between the ages of 18–25, ability to complete the survey in English, and self-identifying as Hispanic. All eligible people were prompted to a consent statement, and the entire study protocol was reviewed and approved by the Institutional Review Board. Participants then completed a variety of questionnaires, including those focused on their demographic identities, acculturative stress, insomnia symptoms, and self-regulated eating behaviors. Questions about demographic information were always asked first, while questions about acculturative stress and insomnia symptoms came next and were presented in a randomized order. Finally, questions about self-regulated eating behavior occurred at the end of the survey. Students participating were incentivized with course credit for their introductory psychology course, upon completion of the survey.

### 2.2. Measures

All measures were selected due to their substantive focus, available validation data in relevant samples (i.e., Hispanic individuals and emerging adults), and brevity. The decision to administer the survey in only English was made due to limited validation of Spanish versions of all measures included in SIESTA and the considerable heterogeneity of Spanish dialects across Hispanic populations.

Demographic Information: Participants self-reported age in years, year at university (i.e., freshman, sophomore, junior, senior, other), family-level socioeconomic status (SES) across four broad groups (poor or low-income, working class, middle class, and rich or upper-class), country of origin, predominate language spoken at home, and height and weight in order to calculate BMI.

Insomnia Symptoms. The Insomnia Severity Index (ISI) is a measure designed to assess both nighttime and daytime insomnia symptoms [31]. The ISI contains 7 items regarding difficulty falling and staying asleep and the satisfaction, distress, and interference associated with sleep problems [31]. A sample item follows: “How NOTICEABLE to others do you think your sleep problem is in terms of impairing the quality of your life?”, with scores ranging from “Not at all Noticeable”, “A Little”, “Somewhat”, “Much”, and “Very Noticeable” [31]. Each item is measured on a 4-point Likert scale. Total scores represent relative insomnia severity with higher scores indicating more severe insomnia symptoms [31]. In the present sample the ISI had a Cronbach’s alpha of 0.856. The ISI has been used in previous studies with both emerging adult and Hispanic populations [32].

Acculturative Stress. The Multidimensional Acculturative Stress Index (MASI) measures stress associated with the pressure to assimilate to Anglo American culture for Hispanic individuals in the US [33]. The MASI contains 36 items related to psychosocial stressors such as English and Spanish language competency, connection to Mexican and American cultures, and perceived stress experienced over the last 3 months [33]. Each item is scored on a 5-point Likert scale from “Not at all stressful” to “Extremely stressful”, following statements like “I feel uncomfortable being around people who only speak English” and “I feel uncomfortable when I have to choose between Latino and American ways of doing things” [33]. Higher total scores indicate higher levels of acculturative stress [33]. The MASI had a high degree of internal reliability in the present study with a Cronbach’s alpha of 0.934. MASI has been used in studies with emerging adult populations [34,35].

Problematic Eating Behavior. The Self-Regulation Eating Behavior Questionnaire (SREB) is a 5-item measure designed to assess self-regulated eating [12]. The SREB begins with three screening items to determine if an individual intends to self-regulate their eating. If no indication to self-regulate is identified, the remainder of the questionnaire is not administered [12]. Sample items include “I give up too easily on my eating intentions” and “I’m good at resisting tempting food”, and are scored on a 5-point Likert scale from “Never” to “Always” [12]. Total scores are calculated by averaging the sum of all items, with higher scores indicating better self-regulated eating behaviors. Previous work has found a mean score of <2.8 indicates low self-regulatory eating behavior [12]. In the present study, the SREB showed acceptable internal consistency, though slightly below the commonly accepted threshold. SREB has been used in previous studies with emerging adults [36].

### 2.3. Analysis

A hierarchical regression was conducted with age, gender identity, BMI, and SES entered in Block 1, and insomnia symptoms in Block 2, to evaluate whether insomnia accounted for additional variance in self-regulated eating behavior. To examine whether acculturative stress helped explain the association between insomnia symptoms and self-regulated eating behavior, a mediation analysis was conducted using bootstrapping procedures with 5000 resamples via the PROCESS macro (version 4.2) for SPSS (version 29) [37] to examine the indirect effect of ISI total scores on SREB means through MASI scores. In this model, ISI scores were specified as the independent variable, MASI scores as the mediator, and SREB means as the dependent variable.

## 3. Results

A total of 523 individuals participated in the Sleep in Emerging Adulthood and Stress Response to Acculturation (SIESTA) study, 482 of which were undergraduate students and 41 were nonstudents and thus excluded from analysis. The final sample included 482 participants comprised mostly of females (70.1% female, 27.0% male) and with a mean age of 18.97 years (*SD* = 1.42). The sample was overwhelmingly US born (88.8%) with 35.5% reporting that they speak only English at home, 18.0% speaking only Spanish at home, and 41.7% spoke both languages at home. The majority of the sample also reported their family’s socioeconomic status as middle class (57.7%). See Table 1 for additional demographic data.

A hierarchical multiple regression was conducted to determine whether insomnia symptoms predicted self-regulated eating behavior in Hispanic emerging adults after accounting for demographic and health-related covariates. The full model explained 10.1% of the variance in self-regulated eating behavior [R^2^ = 0.109, F(5, 476) = 11.69, *p* < 0.001], representing a small effect size (f^2^ = 0.12). To control for the effects of age, gender identity, BMI, and SES, these factors were entered into the first block of the model. In this step, gender identity [t(476) = −3.17, β = −0.142, *p* = 0.002], BMI [t(476) = −4.60, β = −0.205, *p* < 0.001], and SES [t(476) = 2.37, β = 0.104, *p* = 0.018] were significant predictors of self-regulated eating, indicating that women, individuals with higher BMI, and those from lower SES families had lower levels of self-regulated eating behaviors. However, age [t(476) = −0.021, β = −0.001, *p* = 0.984] was not a significant predictor in this model. When insomnia symptoms were entered into the model, they emerged as a significant predictor, t(476) = −4.06, β = −0.179, *p* < 0.001, and its inclusion accounted for a significant increase in explained variance, ΔR^2^ = 0.031, *p* < 0.001, indicating that greater insomnia symptom severity was associated with lower levels of self-regulated eating behavior, as shown in Table 2.

**Figure 1:**
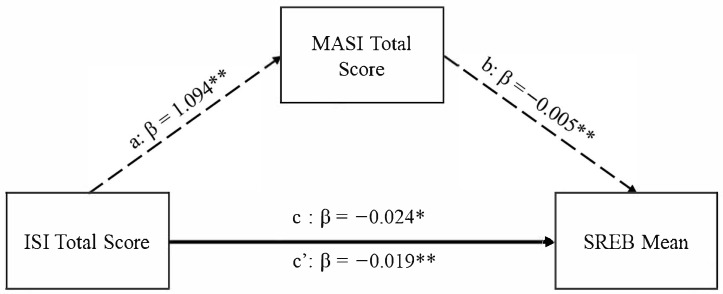
Graphical depiction of acculturative stress mediating the association between insomnia symptoms and self-regulated eating behavior, ** *p* < 0.01, * *p* < 0.05.

The Role of Acculturative Stress: The total effect of insomnia symptoms (i.e., ISI total scores) on self-regulated eating behaviors (i.e., SREB means) was significant (Path *c*, β = −0.024, *p* < 0.01). In addition, the direct effect of insomnia symptoms (ISI total scores) on self-regulated eating behaviors (SREB means) remained significant even after accounting for acculturative stress (MASI scores) (Path *c’*, β = −0.019, *p* < 0.001). Results revealed a positive association between insomnia symptoms (ISI total scores) and acculturative stress (MASI scores) (Path *a*, β = 1.094, *p* < 0.001). In contrast, results revealed a negative association between acculturative stress (MASI scores) and self-regulated eating behaviors (SREB means) (Path *b*, β = −0.005, *p* < 0.001). This model, conducted with 5000 bootstraps, yielded a mean bootstrap estimate of the indirect effect of β = −0.005, 95% CI [−0.029–−0.009], indicating that acculturative stress (MASI scores) partially mediated the effect of insomnia symptoms (ISI total scores) on self-regulated eating behaviors (SREB means). That is, acculturative stress partially explains the association between insomnia symptoms and self-regulated eating behavior. Refer to Figure 1 below.

## 4. Discussion

The current study identified a significant association between higher insomnia symptoms and lower self-regulated eating behavior in Hispanic emerging adults. Such results build on previous work that found insomnia symptoms were significantly associated with uncontrolled and emotional eating in Hispanic adults [16]. Further, acculturative stress partially accounted for the association between insomnia symptoms and self-regulated eating, indicating that acculturative stress may be an important underlying factor in this association. Emerging adulthood is a time marked by significant changes, including potential changes in eating regulation, which may have long-term impacts on an individual’s health as they age [10,11]. Hispanic individuals are at higher risk for negative eating-related health outcomes, including unhealthy BMI, rates of diabetes, and rates of heart disease, all of which are associated with poor eating behavior [38,39]. Notably, low self-regulated eating behaviors have been associated with an increased risk of developing eating disorders and experiencing negative eating-related health outcomes [38,40].

Acculturative stress partially explained the association between insomnia symptoms and self-regulated eating behaviors in Hispanic emerging adults. Previous studies found associations between lower sleep duration and increased acculturative stress [28], while the current study found that increased insomnia symptoms were associated with lower self-regulated eating behavior. Insomnia symptoms have been previously associated with increased stress in undergraduate students and sleep disturbances can decrease an individual’s ability to cope with stress [41]. The present study increases the specificity of these previous studies by identifying culturally unique stress, namely acculturative stress, as an important factor in Hispanic emerging adults. Insomnia symptoms may influence self-regulated eating behavior through reduced executive functioning abilities, particularly reduced inhibition [42]. Additionally, acculturative stress may influence eating behaviors through a complex interplay with guilt, which has been recently found to be associated with problematic eating [43]. Further investigation into specific ways that insomnia and acculturative stress lead to poor self-regulated eating is needed.

Like all studies, the present investigation is not without limitations, including unknown generalizability due to the recruitment strategies, English-only survey administration, and the self-report nature of assessments. Study recruitment was conducted by sourcing participants via required research participation at a large, urban university, which limits the generalizability of the present results to rural, non-academic populations. Another potential limitation of the present study is that the survey was conducted exclusively in English due to the lack of validated translations for all study materials. Future research should consider offering a survey in Spanish to include potential participants who are not English speakers. One notable strength of the current study is the large and diverse sample of Hispanic emerging adults. Future research could explore diet quality, meal timing, and socioeconomic factors related to food access that may have implications for the relationship between sleep and self-regulated eating. Future research might also explore potential differences that may influence sleep and eating patterns across national origin subgroups, and between immigrant and native-born individuals within the Hispanic population. Lastly, as recent work has identified guilt as an important factor in eating disorders [43], the potential interplay between insomnia symptoms, acculturative stress, guilt, and eating behavior appears ripe for inquiry.

## 5. Conclusions

This study demonstrated that greater insomnia symptoms were associated with poorer self-regulated eating and that acculturative stress partially accounted for this association in Hispanic emerging adults. These results build upon previous work that has shown associations between insomnia symptoms and eating behaviors, and they suggest that cultural factors, including acculturative stress, may be an underlying factor in the association between insomnia symptoms and eating behaviors. Future studies investigating how stress is impacted by sleep and, in turn, how that impacts eating behaviors, particularly in underserved communities, may lead to tailored intervention strategies that help prevent illness and promote health and well-being.

## Figures and Tables

**Table 1:** Demographics and descriptive statistics (*n* = 482).

Variables	% (*n*)
Age, mean (SD)	18.97 (1.42)
Gender	
Female	70.1 (338)
Male	27.0 (130)
Other	2.9 (14)
Family socioeconomic class	
Poor or low-income	8.9 (43)
Working class	30.9 (149)
Middle class	57.7 (278)
Rich or upper-class	2.5 (12)
Student classification	
Freshmen	64.3 (310)
Sophomore	17.8 (86)
Junior	11.8 (57)
Senior	5.8 (28)
Other	0.2 (1)
Country of origin	
United States	86.8 (257)
Other country	11.2 (54)
Languages spoken at home	
Only English	35.5 (171)
Only Spanish	18.0 (87)
Both English and Spanish	41.7 (201)
Other languages spoken	4.8 (23)
BMI, mean (SD)	25.07 (5.05)

Note: Age measured in years, all variables self-reported.

**Table 2:** Hierarchical regression predicting self-regulated eating among Hispanic emerging adults.

	Unstandardized		Standardized			
	B	Std. Error	Beta	T	Sig.	95% CI
Block 1						
Age	−0.005	0.020	−0.012	−0.270	0.787	−0.044, 0.033
Gender Identity	−0.213	0.059	−0.164	−3.631	<0.001	−0.329, −0.098
BMI	−0.004	0.001	−0.216	−4.786	<0.001	−0.006, −0.002
SES	0.117	0.041	0.127	2.881	0.004	0.037, 0.198
Block 2						
Age	0.000	0.019	−0.001	−0.021	0.984	−0.039, 0.038
Gender Identity	−0.185	0.058	−0.142	−3.173	0.002	−0.299, −0.070
BMI	−0.004	0.001	−0.205	−4.601	<0.001	−0.005, −0.002
SES	0.096	0.040	0.104	2.372	0.018	−0.016, 0.175
ISI Total Score	−0.020	0.005	−0.179	−4.066	<0.001	−0.030, −0.010

Note. R^2^ = 0.109 for the full model, indicating a small effect size (f^2^ = 0.12).

## Data Availability

The data presented in this study are available on request from the corresponding author due to ongoing internal use.
